# A Case of Chronic Mesenteric Ischemia: Complete Revascularization Using Multiple Procedures

**DOI:** 10.3400/avd.cr.21-00109

**Published:** 2021-12-25

**Authors:** Yusuke Yoshimura, Shun-Ichiro Sakamoto, Atushi Hiromoto, Tomohiro Murata, Kenji Suzuki, Daisuke Yasui, Satoshi Mizutani, Yosuke Ishii

**Affiliations:** 1 Department of Cardiovascular Surgery, Nippon Medical School Musashikosugi Hospital, Kawasaki, Kanagawa, Japan; 2 Department of Radiology, Nippon Medical School Musashikosugi Hospital, Kawasaki, Kanagawa, Japan; 3 Department of Digestive Surgery, Nippon Medical School Musashikosugi Hospital, Kawasaki, Kanagawa, Japan; 4 Department of Cardiovascular Surgery, Nippon Medical School, Tokyo, Japan

**Keywords:** chronic mesenteric ischemia, superior mesenteric artery bypass grafting, median arcuate ligament

## Abstract

Chronic mesenteric ischemia (CMI) involving occlusion and/or stenosis of multiple mesenteric arteries is rare. We report our experience with a 66-year-old man who presented with a more than 3 months history of abdominal pain and vomiting/diarrhea. A diagnosis of CMI due to occlusion of the superior mesenteric artery (SMA) and severe stenosis of the celiac artery by median arcuate ligament syndrome was made. Complete revascularization through iliac artery–SMA bypass grafting and arcuate ligament dissection assisted with staged-catheter intervention successfully alleviated the patient’s symptoms. The patient has maintained a normal daily diet for 6 months postoperatively.

## Introduction

Chronic mesenteric ischemia (CMI) is an abdominal artery-occlusive disease that causes abdominal symptoms due to poor blood supply to the gastrointestinal tract. The etiology of the disease is stenosis or occlusion of independent or multiple mesenteric arteries such as the superior mesenteric artery (SMA), celiac artery (CA), and inferior mesenteric artery (IMA). In a previous report, surgical revascularization or catheter intervention was chosen for treating CMI.^1)^ However, little information about the effectiveness of complete revascularization for CMI involving multiple mesenteric arteries is available.

Here, we report a case of CMI due to SMA occlusion and severe CA stenosis due to median arcuate ligament syndrome (MALS). Complete revascularization using surgical procedures assisted with a catheter intervention successfully alleviated the patient’s symptoms.

## Case Report

A 66-year-old man with a history of paroxysmal atrial fibrillation, heart failure, and cerebral infarction visited a nearby hospital with complaints of abdominal symptoms such as upper abdominal pain, vomiting, and diarrhea lasting for 1 month. He underwent plain computed tomography (CT) and esophagogastroduodenoscopy and was hospitalized under the diagnosis of acute gastroenteritis. During 2 months of hospitalization, the abdominal symptoms recurred every time the patient started taking meals. Contrast-enhanced CT revealed obstruction of the SMA. He was diagnosed with CMI and referred to our hospital to undergo arterial revascularization.

**Figure 1:**
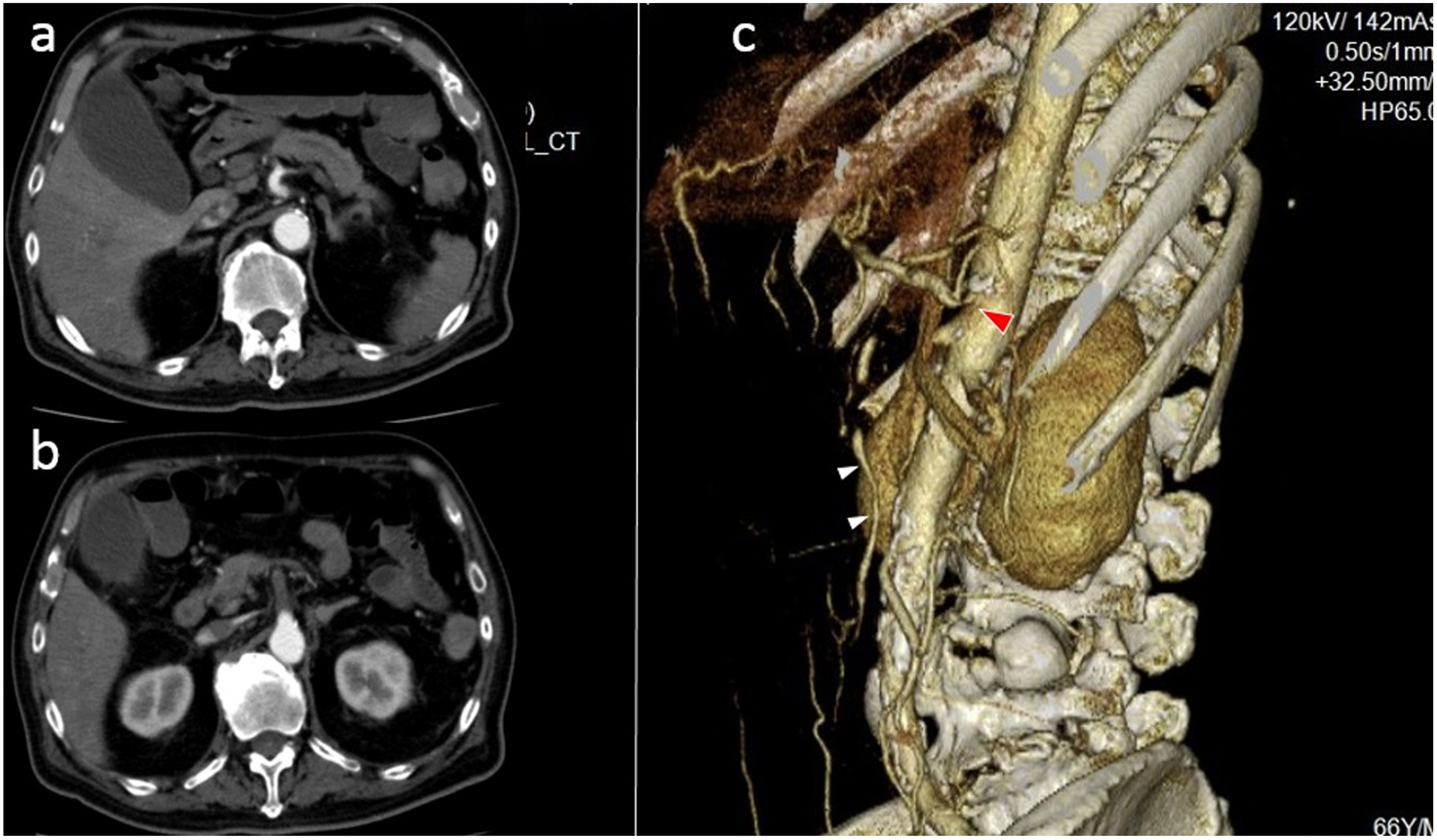
Fig. 1 Preoperative contrast-enhanced computed tomography (CT) images. (**a**) Proximal part of the celiac artery (CA) shows a significant stenosis due to the median arcuate ligament. (**b**) Superior mesenteric artery is occluded in the proximal segment. (**c**) Three-dimensional reconstruction of the CT angiography images represents acute extrinsic stenosis of the CA caused by medial arcuate ligament compression (red arrowhead).

Upon admission to our hospital, the patient was found to have a complication of cholecystitis. According to the results of a colonoscopy conducted at the previous hospital, there were no ischemic findings in the gastrointestinal mucosa. Therefore, laparoscopic cholecystectomy was performed prior to the treatment of CMI. Pathological examination revealed no ischemic findings in the gallbladder wall. His abdominal symptoms were not relieved thereafter; thus, central venous hyperalimentation was required. Abdominal radiography revealed dilatation of the ascending colon, which persisted for more than 1 month. Contrast-enhanced CT revealed severe stenosis in the proximal part of the CA, which was caused by MALS. The SMA was occluded at approximately 5 cm of the proximal segment (**Fig. 1**). Angiography revealed that the CA had a poststenotic dilatation, with delayed flow to its triple branches. The SMA was depicted by the collateral arc of Riolan via the IMA. Based on these findings, which were associated with the patient’s symptoms and the possibility of a thrombosis due to atrial fibrillation, surgical arterial revascularization of both the SMA and CA was indicated. A staged strategy for the removal of the CA lesion was planned, in which the dissection of the median arcuate ligament to release the trunk of the CA was the primary step. Based on postoperative angiography findings, endovascular stent therapy was planned for treating the remaining stenosis of the CA.

**Figure 2:**
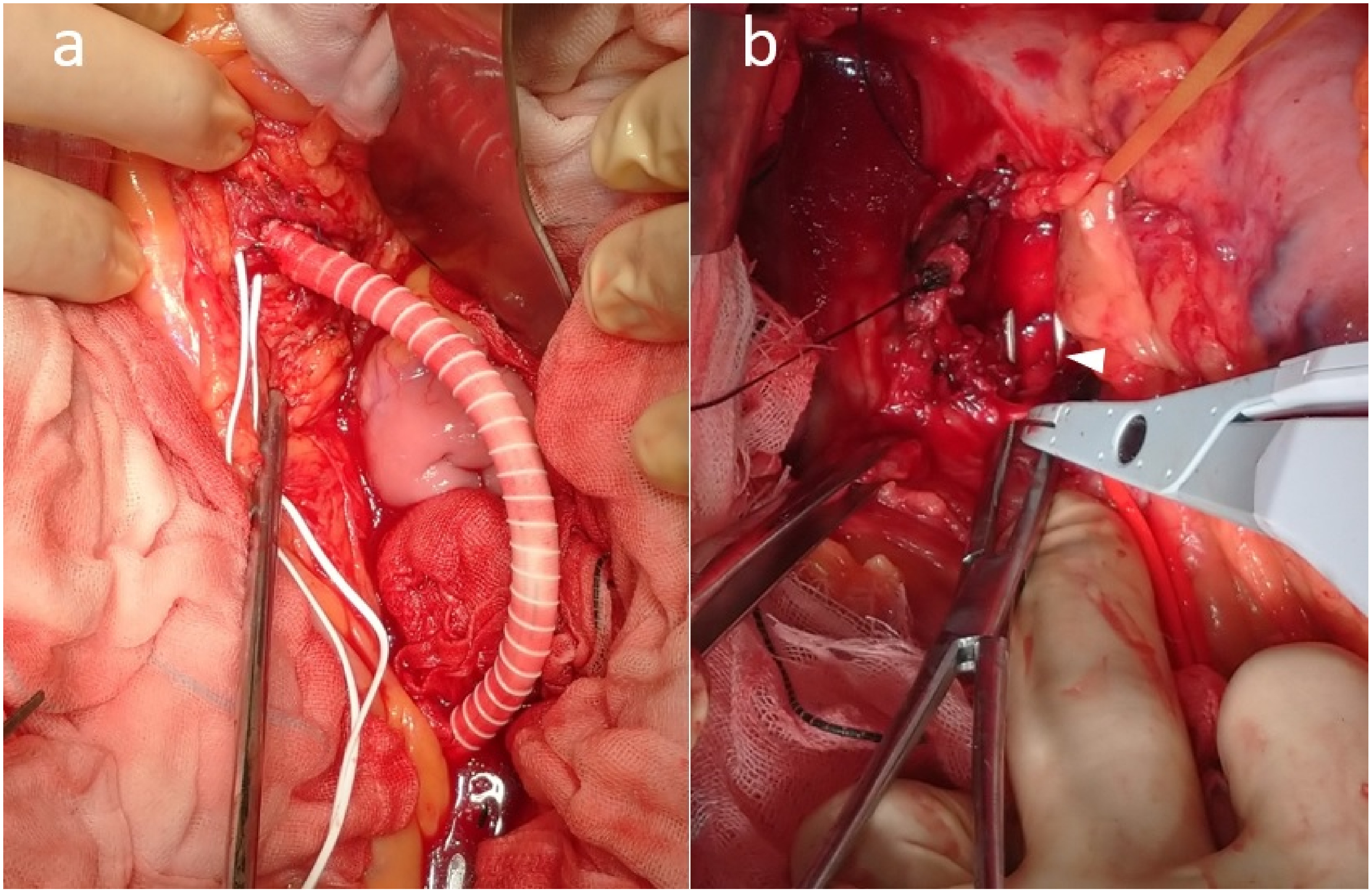
Fig. 2 Surgical revascularization. (**a**) Right external iliac artery–superior mesenteric artery bypass using a 6-mm expanded polytetrafluoroethylene graft. (**b**) Surgical resection of the fibromuscular tissue of the celiac artery (CA). The white arrowhead indicates the root of the CA.

The abdomen was opened via median laparotomy. No ischemic findings were observed in both the small intestine and ascending colon. The ascending colon was dilated with no peristalsis. First, a 6-mm ringed expanded polytetrafluoroethylene (ePTFE) graft was anastomosed to the right external iliac artery. The retroperitoneum was dissected toward the ligament of Treitz. The duodenum was mobilized upward to expose the SMA. The graft was anastomosed to the SMA by using a boat-form vein cuff in C-loop fusion (**Fig. 2a**). Next, the median arcuate ligament was fully dissected over the root of the CA (**Fig. 2b**).

**Figure 3:**
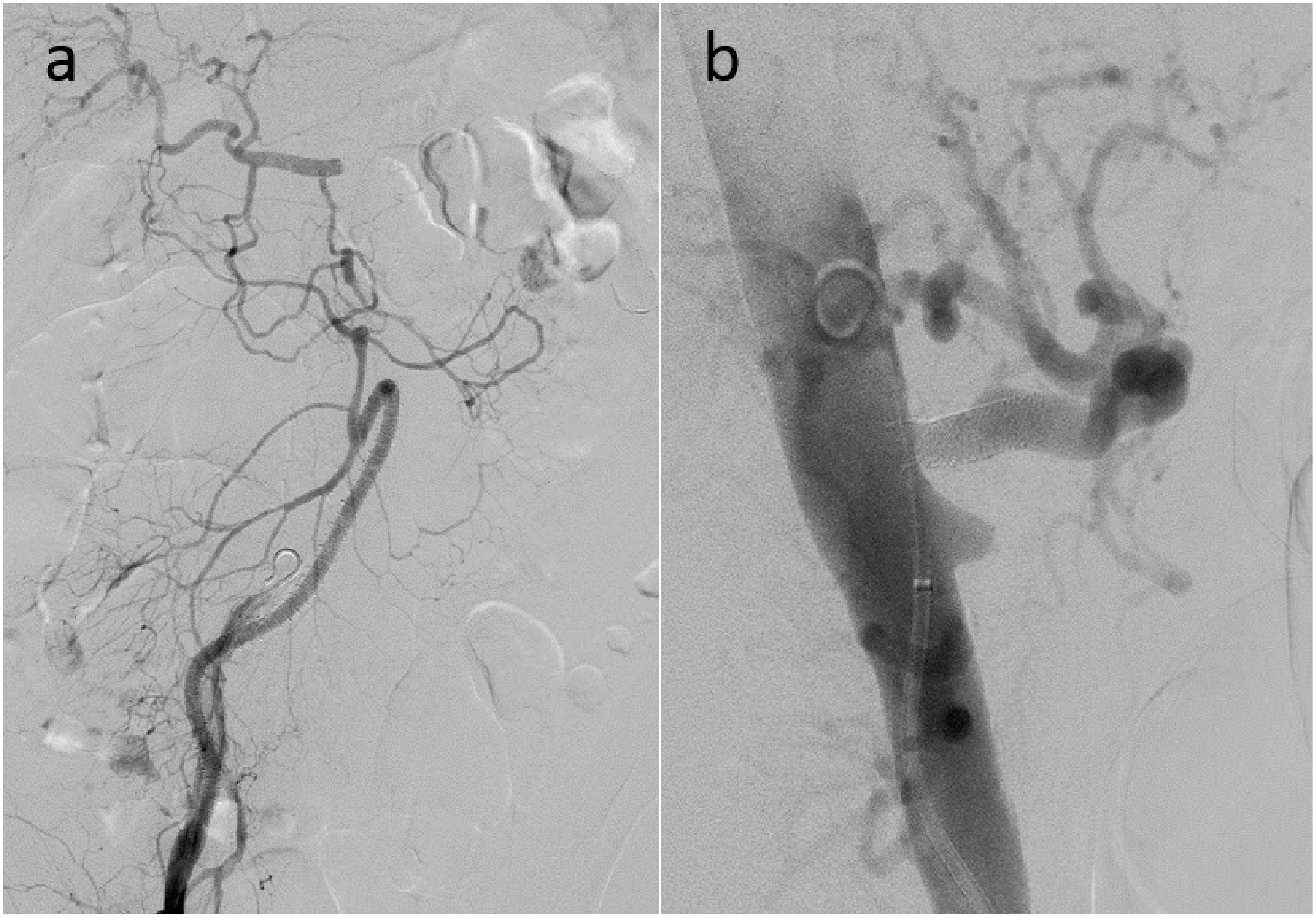
Fig. 3 Postoperative angiogram. (**a**) The bypass graft is patent, providing sufficient flow to the ascending colon and pancreaticoduodenal arterial arcade. (**b**) The celiac artery is expanded with a 7- × 27-mm balloon-expandable stent.

Postoperative angiography revealed that the graft was patent, providing sufficient flow to the ascending colon. In addition, the haptic artery was retrogradely depicted via the pancreaticoduodenal arterial arcade. On the other hand, the ostium of the CA remained stenotic. A balloon-expandable stent (Express LD 7×27 mm; Boston Scientific, Natick, MA, USA) was placed at the stenotic site of the CA, which resulted in good expansion of the CA, with satisfactory flow to the hepatic artery (**Fig. 3**).

The patient recovered bowel movement postoperatively. He had been completely free from abdominal symptoms since the resumption of a regular diet. He was transferred to a rehabilitation hospital owing to disuse syndrome on postoperative day 25 and has maintained a normal daily diet for 6 months postoperatively.

## Discussion

Mesenteric ischemia is a disease that presents abdominal symptoms due to intestinal blood flow being insufficient for the oxygen demand and metabolism in the gastrointestinal tract. CMI is defined as an ischemic symptom that persists for more than 3 months.^2)^ Occlusive disease in mesenteric arteries is the main condition of CMI, whereas symptoms due to single-artery occlusion are uncommon because of the developed collateral network.

The treatment selection for CMI is still controversial. In accordance with the recent guideline from the Society for Vascular Surgery, a meta-analysis that compared endovascular and open revascularizations revealed that endovascular revascularization using a balloon-dilated covered stent was more beneficial in terms of less invasiveness and early outcome. On the other hand, surgical revascularization confers a lower risk of 3-year recurrence.^1)^ The guideline recommends that treatment selection should eventually depend on patient choice and decision making after sufficient explanation by the attending physician.

Regardless of the treatment selection, single-artery revascularization of the SMA was primarily suggested in the treatment of CMI.^1,2)^ In this case, we chose surgical revascularization of both the SMA and CA in consideration of the patient’s conditions. The patient was supposed to transfer to a remote hospital postoperatively to undergo long-term rehabilitation for disuse syndrome. There was concern about symptomatic recurrence associated with bypass graft failure such as thrombosis and thromboembolism due to atrial fibrillation. In addition, revascularization of only the SMA with high-flow bypass can lead to aneurysmal formation or bleeding in the pancreaticoduodenal arterial arch under anticoagulant therapy for atrial fibrillation.^3)^

In this case, complete revascularization of the SMA and CA using bifurcated polyester grafts from the abdominal aorta or common iliac artery was not chosen because of wall thickening and calcification in both arteries.

The external iliac artery on the right side was the only inflow site and a multiple C-loop bypass was required. We considered the possibility of graft elongation and kinking in the long bypass to the CA, although several previous studies reported no notable differences in the graft patencies of antegrade and retrograde bypasses.^4,5)^ Therefore, we independently revascularized CA by treating MALS, and a single bypass to the SMA in a C-loop fashion was selected as the initial treatment. The selection of the graft, vein, or prosthetic remains controversial, without any being superior to the others.^6)^ In this case, because the saphenous vein was not suitable for the graft, prosthetic graft was selected. Distal anastomosis of a 6-mm ePTFE graft to the small SMA tends to cause a discrepancy in the diameter and characteristics of each material, which can result in graft failure. The SMA in our case was small (3.5 mm) and sclerotic. We used the boat-form vein cuff technique in the distal anastomosis to maintain the graft patency.^7)^

In the revascularization of the CA in this case, we initially performed surgical treatment for the MALS, which compressed the celiac trunk with a fibrous band of the diaphragmatic crus surrounding the CA. This pathology is caused by the anatomical background, such as low insertion of the ligament or high take-off CA.^8)^ Angioplasty with stenting in MALS is difficult because the angle of the celiac trunk is too sharp to insert the stent. Surgical dissection of the ligament to release the compression surrounding the celiac trunk is the primary procedure in the treatment of MALS. On the other hand, Reilly et al. reported that treatment for MALS resulted in residual stenosis in more than 50% of the patients.^9)^ Patients with additional reconstruction of the CA are more likely to get relieved from abdominal symptoms than those with release of the median arcuate ligament alone, suggesting reconstruction of residual stenosis of CA even after the treatment of MARS. This finding is compatible with our angiographic finding, suggesting that the CA remained stenotic even after surgical release from extrinsic compression. Therefore, additional revascularization is preferable to prevent the recurrence of symptoms. This suggests that awaiting catheter intervention or one-staged procedure in the hybrid operating room is necessary unless intraoperative verification of CA flow using a Doppler flow meter is available.^10)^ We used the transit-time flow meter to confirm SMA blood flow because there was no probe suitable for the size of CA. Measuring the antegrade flow in one of the CA branches would have been useful to detect residual stenosis in the CA.

## Conclusion

Complete revascularization using surgical and catheter revascularizations might be useful for treating CMI involving multiple mesenteric arteries.
